# Complement Evasion: An Effective Strategy That Parasites Utilize to Survive in the Host

**DOI:** 10.3389/fmicb.2019.00532

**Published:** 2019-03-20

**Authors:** Shuai Shao, Ximeng Sun, Yi Chen, Bin Zhan, Xinping Zhu

**Affiliations:** ^1^Department of Medical Microbiology and Parasitology, School of Basic Medical Sciences, Capital Medical University, Beijing, China; ^2^Department of Pediatrics, National School of Tropical Medicine, Baylor College of Medicine, Houston, TX, United States

**Keywords:** complement system, immune evasion, parasites, complement activation pathways, complement regulatory proteins

## Abstract

Parasitic infections induce host immune responses that eliminate the invading parasites. However, parasites have evolved to develop many strategies to evade host immune attacks and survive in a hostile environment. The complement system acts as the first line of immune defense to eliminate the invading parasites by forming the membrane attack complex (MAC) and promoting an inflammatory reaction on the surface of invading parasites. To date, the complement activation pathway has been precisely delineated; however, the manner in which parasites escape complement attack, as a survival strategy in the host, is not well understood. Increasing evidence has shown that parasites develop sophisticated strategies to escape complement-mediated killing, including (i) recruitment of host complement regulatory proteins on the surface of the parasites to inhibit complement activation; (ii) expression of orthologs of host RCA to inhibit complement activation; and (iii) expression of parasite-encoded proteins, specifically targeting different complement components, to inhibit complement function and formation of the MAC. In this review, we compiled information regarding parasitic abilities to escape host complement attack as a survival strategy in the hostile environment of the host and the mechanisms underlying complement evasion. Effective escape of host complement attack is a crucial step for the survival of parasites within the host. Therefore, those proteins expressed by parasites and involved in the regulation of the complement system have become important targets for the development of drugs and vaccines against parasitic infections.

## Introduction

Parasites are pathogens that live in or on hosts, from which they obtain benefits for their growth, development, and propagation, and thereby cause inevitable harm (Poulin, 2006; Auld and Tinsley, 2015). There are three main classes of parasites that can cause disease in humans, including parasitic protozoa, helminths, and ectoparasites. Parasitic protozoans are single-celled organisms that parasitize the intestinal tract and other tissues, thereby causing various diseases (De Bona et al., 2018). Helminths are multicellular eukaryotic worms, including nematodes, trematodes, and cestodes, which infect billions of people worldwide (Scholte et al., 2018). In addition, severe disease could also be caused by ectoparasites, like *Sarcoptes scabiei*, which burrows into the skin to induce intense itching and the condition known as scabies (Arlian and Morgan, 2017).

Parasitic infections are the most common infections in humans, affecting billions of people worldwide and causing deadly diseases (such as malaria) and often neglected tropical diseases (Hotez et al., 2008). Parasitic infections induce the host immune response, which occurs in an attempt to kill and clear the invasion (Motran et al., 2017; Silva-Barrios and Stager, 2017). The host complement system serves as the first line of defense against parasitic invasion. Complement is a major part of innate immunity that is activated by a robust and efficient proteolytic cascade that eventually results in the opsonization and lysis of many invading pathogens. It is also connected to adaptive immunity and generates inflammatory responses through the production of proinflammatory molecules (Holers, 2014). In addition to its action as an innate and adaptive immunity enhancer in the host’s defense against infection, the complement system plays an alternative role in cell homeostasis by promoting tissue regeneration, morphogenesis, and the coagulation cascade (Kimura et al., 2003; Ricklin et al., 2010). The complement system consists of more than 50 components, including plasma proteins and membrane-bound proteins, some of which serve as PRM that trigger complement activation, and others that act as regulatory proteins that downregulate complement activation to prevent self-damage or autoimmune inflammation (Ricklin et al., 2016). The orchestrated balance between the efficient destruction of pathogens and prevention of unnecessary complement activation in the host tissue is tightly regulated by complement regulatory mechanisms (Ricklin et al., 2016).

To survive within the host, the parasite must overcome the host’s immune response. As a survival strategy, parasites have developed sophisticated mechanisms to escape and defend against complement attack. Parasites express various proteins to effectively play these roles (Morais et al., 2018; Song et al., 2018). Increasing evidence reveals that parasites escape complement attack by using several approaches, including expression of proteins to capture host regulatory proteins; expression of proteins that are homologous to host regulators and interfere with the functions of the host complement system (Jozsi, 2017); and expression of proteins that specifically bind to host complement components and interfere with the final formation of the MAC by inhibiting the classical, lectin, or alternative activation pathways (Braschi and Wilson, 2006; Zhao et al., 2017; Mendes-Sousa et al., 2018; Verma et al., 2018). In this review, we compiled pertinent information regarding parasitic abilities to escape host complement attack as a survival strategy in the hostile environment, and the mechanisms underlying complement evasion.

## Complement System and Activation

**FIGURE 1:**
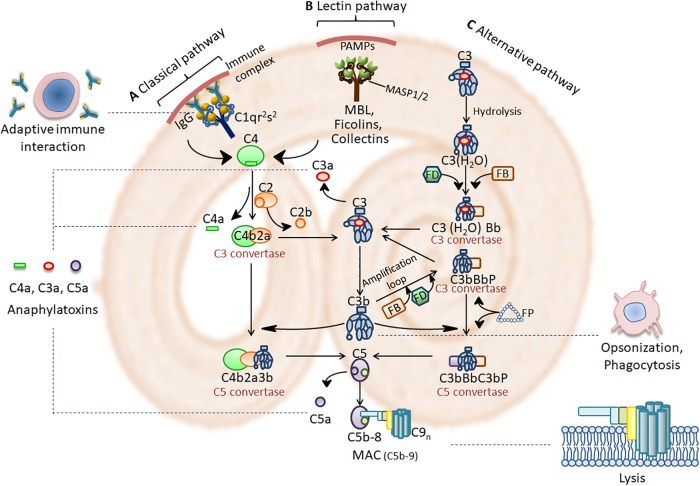
Complement activation cascades and functions. **(A)** Immune complex (IC) activates the classical pathway through activating C1 complex (C1qr^2^s^2^). **(B)** PRMs such as MBL, ficolins and collectins, found in complexes with serine proteases (MASPs), bind to pathogen-associated molecular patterns (PAMPs) on the pathogen surface to activate the lectin pathway. Activation of the classical and lectin pathway leads to cleavage of C4 and C2 to form a C3 convertase (C4b2a). **(C)** The alternative pathway is initiated spontaneously by hydrolyzing C3 into C3(H_2_O) with factors FB, FD and FP. This leads to the formation of C3 convertases of the alternative pathway [C3(H_2_O)Bb or C3bBb]. Complement activation then comes to a core stage that C3 convertase cleave C3 into the anaphylatoxin C3a and the opsonin C3b. C3b then participates in the formation of the classical and lectin pathway C5 convertase (C4b2a3b) and the alternative pathway C5 convertase (C3bBbC3b). C5 convertase cleave C5 into the anaphylatoxin C5a and C5b. Afterwards, C5b assembles with C6, C7, C8, and multiple C9 molecules on the target surface to form MAC (C5b-9). MAC is a 10-nm aperture inserting into the target membrane, which results in the lysis of invading pathogens. The anaphylatoxins C3a and C5a bind to their corresponding receptors, C3aR and C5aR, to mediate inflammation. C3b triggers opsonization which facilitate phagocytic removal of the target. Complement modulates a variety of immune activities and acts as a linker between the native and the adaptive immune response such as augmentation of antibody response and enhancement of immunologic memory.

The complement system in human consists of about 50 molecules that are soluble plasma proteins produced mainly by the liver or membrane-tethered proteins expressed on cell surface, including effector molecules, receptors and regulators (Merle et al., 2015a; Schmidt et al., 2016). Under normal circumstances, complement components remain inactive pro-enzymes or zymogens. On apoptotic cells and the pathogens lacking specific regulators of complement, the complement activation occurs, and the proteases become enzymatically active, resulting in a rapid and efficient cascade (Merle et al., 2015a). Three possible pathways exist for complement activation: the classical pathway, the alternative pathway, and the lectin pathway. Although the initiation steps of the three pathways differ, they converge at the terminal pathway resulting in the lysis of the apoptotic cells and the pathogens (Merle et al., 2015a). The activation cascades are shown in Figure 1.

### Triggering the Classical Pathway

The classical pathway is antibody-dependent and initiated by C1 complex, when binding of C1q to antibody IgM or IgG complexed with antigens (Duncan and Winter, 1988; Gaboriaud et al., 2014; Pednekar et al., 2016). Such binding events cause C1q conformational changes, which lead to the activation of C1r and C1s. Consequently, activated C1s splits C4 and C2, which then form C3 convertase (C4b2a) (Law and Dodds, 1997).

In addition to antigen-antibody immune complexes, C1q can bind directly with certain proteins on the surface of pathogens like bacterial cell walls, or polyanionic structures such as the lipoteichoic acid (Prellner, 1981). The binding of phosphatidylserine of the apoptotic cells, and other agents such as pentraxins (C-reactive protein and pentraxin 3) to C1q also initiates the classical pathway (McGrath et al., 2006; Biro et al., 2007).

### Initiation of the Lectin Pathway

The lectin pathway is activated by microbial sugars instead of immune complexes. It is stimulated when MBL, ficolins (M-ficolin, H-ficolin, and L-ficolin), or collectins (collectin-10 and collectin-11) bind to mannose residues on the surface of the pathogen (Bohlson et al., 2007; Garred et al., 2016). Once bound with glycans, the MASPs become activated from inactive zymogens. A conformational change occurs in MASP-1, following which MASP-2 is cleaved (Garred et al., 2016). Activated MASP-2 then cleaves C4 and C2 to form C3 convertase (C4b2a) (Heja et al., 2012), which continues the cascade as the classical pathway until the formation of the MAC on the membrane of the pathogen. As the lectin pathway can be activated in the absence of antibody, it plays an important role in early infection even before the antibody is generated.

### Initiation of the Alternative Pathway

The alternative pathway is activated by the spontaneous hydrolysis of C3 to from C3(H_2_O). Physiologically, the alternative pathway maintains constitutive activation at low-levels in plasma by a hydrolytic process known as tick-over. This spontaneous hydrolysis of a labile thioester bond converts C3 to a bioactive form C3(H_2_O) (Merle et al., 2015a). This results in a dramatic change in structure that exposes a binding site for FB (Merle et al., 2015a). FB binds to both C3b and C3(H_2_O), which can be cleaved into Ba and Bb by a plasma protease, FD. At this point, C3bBb and C3(H_2_O)Bb are formed as the C3 convertase of the alternative pathway. As C3bBb and C3(H_2_O)Bb are unstable, properdin (factor P, FP) binds with them to stabilize the alternative pathway C3 convertase (Kemper and Hourcade, 2008).

### Generation of C3 Convertase, C5 Convertase and Formation of Activation of the MAC

All three pathways converge at the formation of C3 convertase, which promotes the cleavage of C3 into C3a and C3b. When a large quantity of C3b is generated, C5 convertase is formed by the binding of C3b to C4b2a or C3bBb (C4b2a3b or C3bBbC3b), which marks the activation of the terminal pathway (Schmidt et al., 2016). C5 is cleaved into the potent anaphylatoxin C5a and the bigger split fragment C5b by C5 convertases (Rawal and Pangburn, 2001). Large quantities of C3a and a limited quantity of C5a are released into the fluid phase, and then act on endothelial cells and mast cells (Dias Da Silva and Lepow, 1967; Kohl, 2001; Schraufstatter et al., 2002) as anaphylatoxins because of their ability to induce a shock-like syndrome similar to an allergic reaction (Coulthard and Woodruff, 2015; Hawksworth et al., 2017). The cascade of reactions leading to the assembly of the MAC on the membrane of the pathogen is initiated by C5b. This cascade starts with the binding of C5b to the monomer C6 to form the C5b6 complex, followed by further binding to the monomer C7 to expose the hydrophobic site on C7. The conformationally altered C7 is inserted into the membrane to bind C8 onto itself. Subsequently, C8β binds C5b67, and C8α-γ is inserted into the membrane, leading to the polymerization and insertion of 10–16 molecules of C9 into the membrane to form a pore (Parker and Sodetz, 2002). This 10-nm aperture is formed by the MAC, which disrupts the integrity of the lipid bilayer and allows the transfer of solutes and water across the membrane, resulting in osmotic lysis of the pathogen (Ricklin et al., 2010).

## Regulation of Complement Activation

Activation of the complement system is tightly regulated to prevent uncontrolled amplification of the effects that may cause inflammation or autoimmune diseases (Zipfel and Skerka, 2009; Liszewski and Atkinson, 2015). The first method of regulation is hydrolysis. The complement-activated components that are not bound to the surface of the pathogen, such as C3b and C4b, are rapidly inactivated by hydrolysis. The second method of regulation of complement activation is through a variety of CRPs including RCAs. RCA proteins are the family of RCA gene cluster including complement receptor 1 (CR1 and CD35), membrane cofactor protein (MCP and CD46), decay accelerating factor (DAF and CD55), C4BP, and FH and its alternative splice product Factor H-like 1 (FHL-1) (Schmidt et al., 2016). Other CRPs include C1-INH, FI, CPN1, S-protein, clusterin (SP40/40), CRIg, C8BP, and protectin (CD59) (Noris and Remuzzi, 2013). They target different components to modulate and balance complement activation.

### C1 Inhibitor

To inhibit the initiation stage, C1-INH, a plasma serine protease inhibitor, binds to the active enzymes C1r/s through covalent bond and dissociates it from C1q. C1-INH also binds through covalent bond to MASP-1 and MASP-2 and makes them dissociated from MBL. This leads to limit activation of the classical pathway and lectin pathway (Jiang et al., 2001).

### C3 and C5 Convertase Inhibitors

Negative regulatory proteins that are also present in the plasma and/or on the cell membrane inhibit C3 and C5 convertases (Zipfel and Skerka, 2009). The C4BP has a high binding affinity to C4b and the C4b binding leads to the displacement of C2a from C3 convertase (C4b2a) (Schmidt et al., 2016). FI, an active serine protease in plasma, prevents the formation of C3 convertase and inactivates C4b2a and C3bBb through binding to C3b and C4b with the help of the cofactors FH, C4BP, MCP, and CR1. C3b is cleaved by FI and MCP first into membrane-bound iC3b and C3f, after which C3f is released into the fluid phase. The iC3b is then further cleaved by FI and CR1 into C3dg bound on the surface, and C3c released into the fluid phase. In the alternative pathway, C3bBb is permanently inactivated. Similarly, C4b is inactivated by FI and cleaved into C4c and C4d, thereby inhibiting the formation of C4b2a.

Conformational change in C3b exposes an extended surface in C3b that allows complement regulators FH, DAF, MCP, and CR1 to bind, resulting in the accelerated decay of the alternative pathway C3-convertase and inactivation of C3b (Alcorlo et al., 2015). FH is capable of competing with FB to bind with C3b (Zipfel and Skerka, 2009). DAF binds to C4b and dissociates C2a and Bb from C3 convertases (Fujita et al., 1987). It also displaces Bb from C3bBb to inhibit the formation of C3bBb or C3bBbC3b (Dho et al., 2018). In addition, CRIg can bind with remnant fragments of C3b (C3b, iC3b, and C3c) on the cell membrane to inhibit the alternative pathway (Zipfel and Skerka, 2009).

### MAC Inhibitors

At the stage of MAC formation, several inhibitory proteins prevent the insertion of the MAC into the lipid bilayer. The S-protein, also known as vitronectin, binds with C5b67, C5b-8, and C5b-9 to inhibit MAC insertion. The SP40/40 not only binds directly to the C5b-9 complex but serves as a cofactor of the S-protein. Both C8BP and CD59 inhibit the binding of C9 to C5b-8, and C9 polymerization (Zipfel and Skerka, 2009).

Thus, regulation of complement activation at various stages of all three pathways spontaneously maintains homeostasis. Otherwise, any breaks in this balance could lead to autoimmune syndromes, such as atypical hemolytic uremic syndrome, with clinical features of damaged red blood cells and platelets, and kidney inflammation due to malfunction of the complement system (Yoshida et al., 2018).

## Biological and Alternative Functions of Complement Activation

### Complement Is Activated to Eliminate Pathogens by MAC or Opsonization

When complement is activated, it contributes to the defense against pathogens, which is not only evolved as part of the innate immune system but remains a link to the adaptive immune system. Complement destroys pathogens directly via the MAC. Once complement activation has been initiated, all three pathways lead to the formation of C3 convertase, which initiates a cascade of enzymatic reactions that eventually lead to the formation of the MAC on the surface of pathogens, resulting in osmotic lysis of invading bacteria (especially Gram-negative bacteria), viruses, and parasites (Merle et al., 2015b).

Moreover, complement fragment C5a recruits neutrophils, monocytes, and macrophages to clear pathogens through a process known as opsonization (Ricklin et al., 2010; Martin and Blom, 2016). The C3b and C4b bind with CR1 expressed on phagocytes and erythrocytes to enhance IgG-mediated phagocytosis or clear soluble immune complexes (Freeley et al., 2016). In addition, iC3b can be specifically recognized by CR3 and CR4 on monocytes and neutrophils (Wang et al., 2015; Lubbers et al., 2017). This causes the phagocytes to release toxic reactive oxygen compounds and microbicidal components, such as lysozyme and proteases that kill pathogens (Martin and Blom, 2016).

### Alternative Functions of Activated Complement Components

Besides the participation of complement in innate immune defense, some of the original complement proteins, or intermediate components generated during activation, have many other immunity-enhancing functions. In addition to initiating the classical activation of complement, C1q has many unconventional functions. It is noteworthy that apart from its role as part of the complement system, C1q recognizes its receptors expressed on phagocytes, such as neutrophils and macrophages, and stimulates these phagocytes to release reactive oxygen species, such as H_2_O_2_ and superoxide, to attack pathogens. Furthermore, C1q plays a role in modulating dendritic cell (DC) maturation (Son et al., 2012), B cell tolerogenic capacity, and IgM or IgG production (Young et al., 1991). Moreover, C1q is reportedly involved in the clearance of apoptotic cells via CD91, which is a multi-protein-binding scavenger receptor complex containing CRT (Ogden et al., 2001).

Anaphylatoxins C3a and C5a stimulate DCs to express the major histocompatibility complex II (MHC-II) and CD86 via C3a and C5a receptors, thereby promoting their maturation as APCs (Peng et al., 2008, 2009). Locally produced C3a and C5a also interact with their receptors on T lymphocytes involved in maintaining T cell proliferation and differentiation (Strainic et al., 2008). The interaction between C3d and complement receptor CR2 on B cells during antigen-induced activation promotes B cell activation and facilitates the transformation of naïve B cells into antibody-producing effector and memory B cells (van den Elsen and Isenman, 2011; Donius et al., 2013). These alternative functions of complement components bridge the innate immune response with the adaptive immune response to coordinate the elimination of invading pathogens. The functions of the complement system are shown in Figure 1.

## Complement Evasion by Parasites

Over millions of years of evolution, parasites, including parasitic protozoans, helminths and ectoparasites, have developed sophisticated mechanisms as strategies of survival to escape immune attack in the host. The first strategy developed was the inhibition of complement activation as the initial step to evade host immune clearance, particularly at the early stages of invasion. Complement plays an important role in the defense against parasitic infections, which relies on its direct lysis on invading parasites through the formation of the MAC, and acts as a bridge to adaptive immune responses. Parasites have evolved to develop several mechanisms that inhibit or interrupt the functions of host complement as the first steps to escape host immune attack.

### Recruitment of Host Regulatory Proteins to Inhibit Complement Activation

The C1-INH is a soluble regulator of complement activation that negatively regulates the classical and lectin pathways by inhibiting C1r, C1s, MASP-1, and MASP-2, the activating proteases of the complement cascade. The intracellular protozoa, *Plasmodium falciparum*, which causes the deadliest malaria in humans (Rich et al., 2009), could recruit and utilize C1-INH to inhibit complement activation (Mejia et al., 2016). Merozoites, the invasive form of blood-stage malarial parasites, actively recruit C1-INH to their surface when exposed to human serum. A member of the merozoite surface protein 3 family, PfMSP3.1, worked as a direct interactive partner to bind with C1-INH, to inhibit C1s, MASP1, and MASP2 (Kennedy and Wijeyewickrema, 2017).

Host complement FH, a CRP, is the main soluble inhibitor of the alternative pathway against the formation of C3-convertase. It achieves this inhibition by supporting the conversion of C3b to iC3b (Zipfel and Skerka, 2009). *Echinococcus granulosus*, a cestode that causes cystic echinococcosis in humans, expresses a protein that sequestrates FH on the hydatid cyst wall to inhibit complement C3b deposition (Diaz et al., 1997; Breijo et al., 2008). The binding molecule remained unknown until further research showed that it was InsP6, a major component of the acellular laminated layer (LL) of the hydatid cyst wall, that was bound with FH to inhibit the alternative pathway (Irigoin et al., 2008). Mosquito midgut epithelial cells also express two proteins (40 and 100 kDa) as receptors that captured FH to inhibit the deposition of C3b and impair activation of the alternative complement pathway (Khattab et al., 2015).

Decay-accelerating factor (DAF; CD55), another human complement regulatory factor, causes the accelerated decay of C3 and C5 convertases by associating with C4b and C3b deposited on the cell membrane (Fujita et al., 1987; Heine et al., 2003). *Schistosoma mansoni* is a blood fluke that causes intestinal schistosomiasis. When incubated with normal human erythrocytes, but not with DAF-deficient erythrocytes, *S. mansoni* became resistant to complement lysis *in vitro* (Horta and Ramalho-Pinto, 1991). Further study showed that *S. mansoni* acquired DAF from host erythrocytes via the expression of a GPI anchor on the surface of the worm (Ramalho-Pinto et al., 1992). The ability of the trypsin-treated *S. mansoni* worm to acquire DAF was reduced (Ramalho-Pinto et al., 1992). Treatment with GPI-specific phospholipase D (GPI-PLD) facilitated the binding of DAF to the surface of the schistosomula (Carvalho et al., 1994).

As a membrane-bound inhibitor of the cytolytic MAC, CD59 reduces C9 polymerization on the cell surface by binding to C8α and C9 (Venneker and Asghar, 1992). The N-linked glycosylation of CD59 is related to its complement-inhibitory activity (Ninomiya et al., 1992). *P. falciparum* is able to acquire the intrinsic host factor, CD59, to restrict complement attack on the infected erythrocyte (Wiesner et al., 1997). Furthermore, *P. falciparum* expressed mannosyltransferase (PfPIG-M), which is involved in GPI synthesis, and thereafter increased the levels of the GPI-anchored protein, CD59, on the cells, indicating that the GPI anchor is involved in the capture of CD59 on the surface of *P. falciparum*-infected erythrocytes (Kim and Hong, 2007). *Trichomonas vaginalis*, an anaerobic flagellated protozoan parasite, also acquired CD59 from different host cells, including red blood cells, during infection, to protect the parasite from being lysed by host complement (Ibanez-Escribano et al., 2015).

### Expression of Homologous Proteins With Host Regulators of Complement Activation

To avoid complement-mediated lysis, some parasites express a variety of CRPs on their surfaces. As more parasite genomes have been deciphered, parasitic helminths have been shown to express many mammalian-like receptors for host growth factors, cytokines, or hormones to regulate the growth, development, signaling, and reproduction of the parasite (Hu et al., 2003). More results suggest that parasites modulate host anti-parasite immune responses by expressing host immunological inhibitors and receptors of some immunological components (Ramirez-Toloza and Ferreira, 2017). The antigenic similarity between host- and parasite-expressed proteins may mask the host’s immune system to recognize invading parasites, and thus protect the parasite from elimination (Abu-Shakra et al., 1999).

Parasites express orthologs of host complement components or regulators to modulate or inhibit the functions of the host complement. *S. japonicum*, a blood fluke that causes Asian schistosomiasis, expresses protein (Schistosome CRIT) that share similarities with host complement C2 receptor inhibitor trispanning (CRIT) (Inal, 2005). This expression is indicative of their involvement in host complement activation or regulation. Schistosome CRIT is located on the surface tegument of the *Schistosoma* parasite and enables it to bind C2 via its extracellular domain. It subsequently inhibits the binding of C2 to C4b, to interfere with the formation of C3 convertase (C4b2a). The CRIT is an example of molecular mimicry, as it reportedly binds C2 with a domain that is homologous to one region of human C4b. Both the classical and lectin complement pathways are interrupted when C2 is hijacked (Cestari Idos et al., 2009). The C2 binding site of schistosome CRIT is located at an 11-amino acid sequence at the C-terminus of the first extracellular domain, which is involved in the inhibition of the classical complement pathway and reduction of immune complex-mediated inflammation (Inal et al., 2003). *Trypanosoma cruzi*, an intracellular protozoan parasite that causes Chagas disease, also expresses CRIT on the surface of trypomastigotes to inhibit C2-associated complement activation (Ramirez-Toloza and Ferreira, 2017).

In addition to the C2 receptor inhibitor, adult schistosomes express the C3 receptor on their surface tegument. During complement activation, C3 binds to the worm’s surface through the C3 receptor and stimulates the replacement of the outer tegument, which is shed during complement attack (Silva et al., 1993). Through this expression of the C3 receptor and shedding of the C3–C3 receptor, parasites are able to consume C3 from serum, and consequently become non-activators of the alternative pathway (Marikovsky et al., 1986; Rasmussen and Kemp, 1987).

In addition to its recruitment of host DAF on its surface to avoid host complement lysis, the *T. cruzi* trypomastigote also expresses DAF (T-DAF) on the surface of its virulent forms to inhibit complement activation by blocking C3, similar to mammalian DAF (Joiner et al., 1988; Kipnis et al., 1988; Tambourgi et al., 1993). Further studies have demonstrated that *T. cruzi* expressed a 160 kDa (GP160) complement regulatory glycoprotein on the surface of trypomastigotes (Norris et al., 1989). The gp160 gene was verified to share significant DNA sequence homologous with the human DAF gene (Norris et al., 1991). GP160 can inhibit the formation of the alternative and classical C3 convertase as it is a member of the C3/C4 binding family of complement regulators. This prevents the activation and amplification of the complement cascade on the parasite’s surface (Norris and Schrimpf, 1994; Norris et al., 1997).

An earlier study described a schistosome complement inhibitor, a 94-kD protein of *S. mansoni* (SCIP-1), expressed on the surface of *S. mansoni* larvae and adults, which was found to be functionally and antigenically related to human CD59. It binds to human C8 and C9, and inhibits the assembly of C5b-9 (Parizade et al., 1994). In addition, other CD59 homologs have been identified in the schistosome genome displaying the consensus CCXXXCN sequence at the C terminus (Wilson and Coulson, 2009) and in the membrane fraction of the live schistosome tegument (Castro-Borges et al., 2011). CD59 homologs (FhCD59-1,2,3) have also been found on the surface tegument of the trematode, *Fasciola hepatica*, a liver fluke (Shi et al., 2014). FhCD59-2 showed a phylogenetic relationship with SmCD59-2 on the surface tegument of *S. mansoni* (Shi et al., 2014). However, analogs of mammalian cell-expressed recombinant schistosome CD59 showed no inhibition of complement activity *in vitro*, which possibly differs from the action of native proteins expressed in the tegument (Farias et al., 2013). And both the location and potential function of the CD59-like proteins in *F. hepatica* even require further biochemical analyses to elucidate.

### Expression of Proteins to Inhibit Host Complement Activation

**FIGURE 2:**
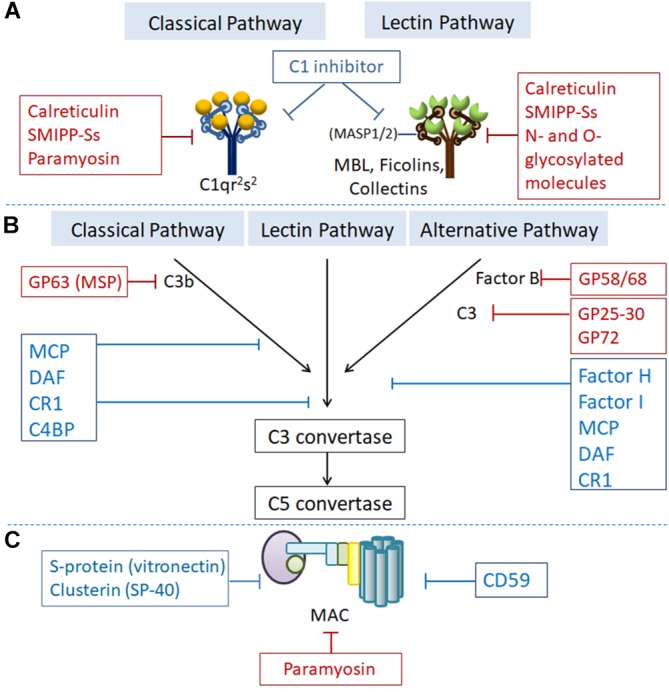
Regulation of complement activation by RCAs or parasite-expressed proteins targeting different complement components at the different steps of complement activation. Proteins showed in the blue boxes are human complement regulatory proteins. Proteins showed in the red boxes are parasites-generated to inhibit complement activation. **(A)** At the initiation step, C1 inhibitor binds to the active enzymes C1r/s and MASP-2 and dissociates them from C1q and MBL, respectively. Calreticulin, paramyosin, SMIPP-Ss, and *N*- and *O*-glycosylated molecules expressed by parasitic protozoa or helminths bind to C1q, MBL and ficolins to inhibit initiation of the classical and lectin pathway. **(B)** C3 convertase (C3bBb) undergoes an accelerated decay mediated by complement receptor 1 (CR1), decay accelerating factor (DAF), C4b-binding protein (C4BP) or factor H (FH). C3b is inactivated to iC3b by factor I (FI) with cofactor CR1, FH, C4BP or membrane cofactor protein (MCP). GP63 expressed by *Leishmania* binds to C3 leading to proteolysis of the active component C3b to form the inactive iC3b, thus preventing the formation of C3-convertase. GP58/68 expressed by *T. cruzi* binds with FB and interferes the formation of C3 convertase to inhibit alternative pathway. **(C)** Under normal conditions, CD59, S-protein (vitronectin), and Clusterin (SP-40) prevent the formation of the MAC. Paramyosin generated by helminth like *T. spiralis* bind with C8 and C9 to inhibit the formation of MAC.

In addition to their expression of parasite-encoded regulators, which mimic host complement regulators, to inhibit complement activation, parasites also express or secrete a variety of proteins that directly bind to some complement components to inhibit their activation by targeting various stages (Figure 2).

#### Expression of Molecules That Inhibit C1q and MBL

The initiator of the classical complement pathway, C1q, plays an important role both in complement activation and in the activation of some immune cells by binding to the C1q receptor on their surfaces. CRT is a calcium-binding protein with a broad range of functions associated with immunomodulation (Malhotra et al., 1990). It contains the globular N-domain, proline-rich P-domain, and acidic C-domain, which can bind to C1q, resulting in the inhibition of C1q-dependent complement activity (Boelt et al., 2016). Studies have shown that CRTs are expressed on the surfaces of several parasites, such as *Trypanosoma carassii, Necator americanus, Trichinella spiralis*, and *Brugia malayi*, where it acts as an inhibitor of C1q, to facilitate the survival of the parasites in the hosts (Pritchard et al., 1999; Oladiran and Belosevic, 2010; Yadav et al., 2014; Zhao et al., 2017).

The tissue-dwelling nematode, *T. spiralis*, which causes serious trichinellosis in humans, expresses CRT (Ts-CRT) that can bind to C1q to inhibit the activation of the classical complement pathway. This results in the failure of MAC generation on the parasites, thereby conferring protection from the attack by the activated complement (Zhao et al., 2017). Binding of Ts-CRT to C1q also inhibited C1q involved pathogen-clearance functions, such as reduced recruitment of immune effect cells (neutrophils, eosinophils, and macrophages) to the site of parasitic infection and reduced the release of reactive oxygen intermediates (ROIs) and reactive nitrogen intermediates (RNIs) (Zhao et al., 2017).

The CRT expressed by *B. malayi*, a nematode that causes lymphatic filariasis in humans, could bind to human C1q. Bm-CRT inhibited C1q-dependent lysis of immunoglobulin-sensitized red blood cells (Yadav et al., 2017). Molecular docking has identified that interactions between Bm-CRT and C1q occurred at the site of the C1qB chain (IgG/M and CRP binding sites on C1q), and Bm-CRT conserved and non-conserved regions of the N/P-domain, in which 37 amino acids were involved in the interaction (Yadav et al., 2014, 2017). Mapping of the C1q binding site in the Hc-CRT secreted by *H. contortus* has shown that the N-terminal sequences of GKYYDDAKRD and AKFPKKFT were involved in the binding of C1q and suppression of complement-mediated cell lysis (Naresha et al., 2009).

*Taenia solium*, a tapeworm in the intestine of pigs that causes cysticercosis in humans, secreted CRT bound to C1 and induced a dose-dependent inhibition of C1-initiated complement activation (Laclette et al., 1992). More studies have shown that the *S. scabiei* secreted proteolytically inactive serine protease paralogs (SMIPP-Ss) that are bound both to C1q to inhibit the classical pathway, and to FP resulting in assembly failure of the alternative pathway convertases and thereby inhibiting the alternative pathway (Bergstrom et al., 2009).

*Haemonchus contortus*, a gastrointestinal nematode that parasitizes domestic animals, expresses GAPDH, one of the components of *H. contortus* ES products. The parasite enzyme trapped C1q and inhibited the complement-mediated lysis of sensitized sheep erythrocytes. The C1q binding site of Hc-GAPDH was mapped to the N-terminal (Vedamurthy et al., 2015).

*Trypanosoma cruzi* utilizes *N*- and *O*-glycosylated molecules on the surface of trypomastigotes and amastigotes to bind with L- and, H-ficolins, and MBL (Kahn et al., 1996). This results in the failure of MASP-2-induced C2 and C4 cleavage (Cestari and Ramirez, 2010; Ribeiro et al., 2015). Similarly, CRT of *T. cruzi* could bind to the collagenous portion of L-ficolin to inhibit the activation of the lectin pathway (Sosoniuk et al., 2014). Tc-CRT also bound MBL thus inactivating the lectin pathway (Lidani et al., 2017; Sosoniuk-Roche et al., 2017). Two scabies mite proteins, SMIPP-S D1 and I1, were also shown to be capable of binding with MBL to inhibit activation of the MBL-initiated lectin pathway (Reynolds et al., 2014).

One more important C1-INH is the helminth-secreted Pmy, which plays an important role in immunomodulatory functions by evading complement-mediated lysis. *T. spiralis* and *S. mansoni* express Pmy on their surfaces, which is also able to bind to C1q to inhibit C1q-initiated complement activation (Deng et al., 2007; Zhao et al., 2014).

#### Inhibition Occurs at the C3 and C5 Convertase Steps

Formation of the C3 and C5 convertases is a critical step for complement activation. Inhibitors bind to C3 and C5 convertases and interfere with their enzymatic reactions, thereby inhibiting the final formation of the MAC. Inhibition of C3 and C5 convertases also inhibits the release of anaphylatoxin C5a and the C5a-mediated recruitment of neutrophils.

During *T. cruzi* infection in mammals, the infective trypomastigote becomes resistant to lysis induced by the alternative complement pathway. The majority of C3 on the trypomastigote is in the inactive form, iC3b, which fails to form C5 convertase (Joiner et al., 1986). *T. cruzi* express the C3 acceptor in the infective trypomastigote stage and is a molecule of 25–30 kDa, whereas the C3 acceptor in the epimastigote stage is GP72 to inhibit the formation of alternative pathway C3 convertase (Joiner et al., 1986). A glycoprotein expressed on the surface of the trypomastigote, with molecular weight of 58/68 kDa (Gp 58/68), is another CRP that inhibits the formation of cell-bound and fluid phase alternative pathway C3 convertase, possibly through its interaction with FB rather than C3b (Fischer et al., 1988). Further investigation with recombinant protein demonstrated that this protein binds to human complements C3b and C4b to inhibit the activation of the complement cascade (Beucher et al., 2003).

Hc-GAPDH, which inhibits the C1q-initiated classical complement pathway, also could act as a C3 binding protein (C3BP) to inhibit complement activation, as measured by the hemolytic assay and MAC formation (Sahoo et al., 2013). The binding of C3BP to C3 through its N-terminal domain resulted in the inhibition of complement activation (Vedamurthy et al., 2015).

The GP63 expressed by *Leishmania*, an intracellular protozoa that causes leishmaniasis, is the most abundant surface glycoprotein on *Leishmania* promastigotes (Yao et al., 2003) that binds to C3 with high affinity, leading to proteolysis of the active component C3b to form the inactive iC3b, thus preventing the formation of C3-convertase (Yao, 2010).

#### MAC Formation Is Impeded

The terminal complement pathway is the final cytolytic step in the complement cascade, which results in formation of the MAC, a lytic assembly of C5b, C6, C7, C8, and multiple molecules of C9. In addition to the expression of host CD59 homologs on their surfaces to inhibit formation of the MAC and directly restrict complement-mediated lysis, parasites also express several other molecules that interfere with MAC formation as a final measure of protection against complement attack.

*Trichinella spiralis* expressed paramyosin (Ts-Pmy) on the surface of larvae and adult worms acts as an important immunomodulatory protein by not only binding to C1q to inhibit the initiating step of classical complement activation (Zhang et al., 2011; Sun et al., 2015), but binding to C8, C9 to interfere with MAC formation, and thus protecting the parasite from complement-mediated killing (Hao et al., 2014). Mapping of the C8/9 complement binding site has been narrowed down to 14 amino acid residues at the C-terminus (^866^Val-^879^Met) of Ts-Pmy, which inhibited C9 polymerization and complement-mediated lysis of rabbit erythrocytes (Zhao et al., 2014). The similar C8/C9 binding sites can also be found in *S. mansoni*-Pmy, located within the amino acid sequence of ^744^Asp–^866^Met at the C-terminus (Deng et al., 2007). The identification of complement binding sites on CRPs is important to gain a better understanding of their inhibitory mechanisms, as it relates to the design of drugs and vaccines against parasitic infections.

The host CRPs used by parasites and the parasite-generated CRPs described in this review are listed in Table 1.

**Table 1:** A selection of host complement regulatory proteins utilized by parasite or parasite-produced complement regulatory proteins described in this review.

Recruitment of host regulatory proteins to inhibit complement activation				
**Proteins**	**Species**	**Ligands**	**Effects**	**Reference**

PfMSP3.1	*Plasmodium falciparum*	C1-INH	To recruit and utilize C1-INH to inactivate C1s and MASP-2	Kennedy and Wijeyewickrema, 2017
InsP6	*Echinococcus granulosus*	FH	To recruit and utilize FH to inactivate the conversion of C3b to iC3b	Irigoin et al., 2008
Proteins (40 and 100 kDa)	Mosquito midgut epithelial cells	FH	To capture FH to inhibit the deposition of C3b and impair activation of the alternative complement pathway	Khattab et al., 2015
A GPI anchor on the surface of the worm	*Schistosoma mansoni*	DAF	To acquire DAF from host erythrocytes to form a complex with C4b and C3b deposited on the cell membrane causing accelerated decay of C3 and C5 convertases	Ramalho-Pinto et al., 1992
PfPIG-M	*Plasmodium falciparum*	CD59	To acquire the intrinsic CD59 to reduce C9 polymerization on the cell surface by binding to C8α and C9	Kim and Hong, 2007
Unknown	*Trichomonas vaginalis*	CD59	To acquire CD59 from different host cells, including red blood cells to reduce C9 polymerization	Ibanez-Escribano et al., 2015

**Expression of** **homologous proteins with host regulators of complement activation**				

**Proteins**	**Species**	**Ligands**	**Effects**	**Reference**

Complement Receptor Inhibitor Trispanning (CRIT)	*Schistosoma japonicum Trypanosoma cruzi*	C2	To be homologous with host CRIT; To hijack C2 to interrupt the classical and lectin complement pathways	Cestari Idos et al., 2009; Ramirez-Toloza and Ferreira, 2017
*Schistosoma mansoni* C3 receptor	*Schistosom mansoni*	C3	To be homologous with host C3 receptor; To consume C3 from serum, and consequently become non-activators of the alternative pathway	Silva et al., 1993
T-DAF (GP160)	*Trypanosoma cruzi*	C3 C4	To be homologous with host DAF; To inhibit complement activation by blocking C3 and C4	Norris et al., 1991, 1997; Tambourgi et al., 1993; Norris, 1998
SCIP-1	*Schistosom mansoni*	C8 C9	To be homologous with host CD59; To bind to human C8 and C9 and inhibit the assembly of C5b-9	Parizade et al., 1994

**Expression of proteins to inhibit host complement activation**				

**Proteins**	**Species**	**Ligands**	**Effects**	**Reference**

Calreticulin	*Trichinella spiralis Trypanosoma carassii Necator americanus Brugia malayi Trypanosoma cruzi*	C1q L-Ficolin MBL	To inhibit activation of the classical and lectin complement pathway	Pritchard et al., 1999; Oladiran and Belosevic, 2010; Sosoniuk et al., 2014; Yadav et al., 2014; Sosoniuk-Roche et al., 2017; Zhao et al., 2017
Paramyosin	*Trichinella spiralis Schistosom mansoni*	C1q C8 C9	To inhibit activation of the classical complement pathway and the formation of MAC	Deng et al., 2007; Zhang et al., 2011; Zhao et al., 2014
SMIPP-Ss	*Sarcoptes scabiei*	C1q FP	To inhibit activation of the classical and alternative pathway	Bergstrom et al., 2009
SMIPP-S D1 and I1	*Sarcoptes scabiei*	MBL	To inhibit activation of the lectin complement pathway	Reynolds et al., 2014
*N*- and *O*-glycosylated molecules	*Trypanosoma cruzi*	L-Ficolin H-Ficolin	To inhibit activation of the lectin complement pathway	Kahn et al., 1996
Glyceraldehyde-3-phosphate dehydrogenase (GAPDH)	*Haemonchus contortus*	C1q C3	To inhibit haemolysis and MAC formation	Sahoo et al., 2013; Vedamurthy et al., 2015
GP58/68	*Trypanosoma cruzi*	FB	To bind with FB and inhibit alternative pathway	Fischer et al., 1988
GP63	*Leishmania mexicana*	C3b	To form iC3b	Yao et al., 2003
Protein (25–30 kDa) (trypomastigote) GP72 (epimastigote)	*Trypanosoma cruzi*	C3	To inhibit the formation of the C3 convertase in the alternative pathway	Joiner et al., 1986


## Parasite-Generated Complement Regulatory Proteins as Vaccine and Drug Targets

Parasite-generated complement inhibitory proteins are actively involved in the inhibition of host complement activation and are important for the survival of parasites within the host. Therefore, these proteins have emerged as prime targets for the development of drugs and vaccines against infections, or regulation of disease progression. Immunization with these proteins may reduce the defensive ability of the parasites against host complement attack, thereby rendering them more susceptible to the host’s immune defense, and ultimately leading to their expulsion from the host. Immunization with C4BP-fused MSP1_19_ induced protective immunity in BALB/c mice against the otherwise lethal malarial parasitic challenge of *Plasmodium yoelii*, possibly through protection of the parasite from complement lysis (Ogun et al., 2008). Vaccination with complement regulator CRIT ed1 synthetic peptide conferred protection against the challenge of *Schistosoma japonicum* in mice through the inhibition of complement activity both *in vitro* and *in vivo* (Ma et al., 2017). *T. cruzi* expresses a complement regulatory protein (Tc-CRP) as a major antigen that induces the production of lytic antibodies during *T. cruzi* infections, making it a potential target for vaccine development (Henrique et al., 2016).

As an important CRP, Pmy has become another target of interest for the development of vaccines against various helminth infections. Mice immunized with native or recombinant Pmy of *S. mansoni* show a significant reduction in worm burden when challenged with *S. mansoni* cercariae (Pearce et al., 1988). The monoclonal antibody, 9G3, which targets the complement binding site of Ts-Pmy, could partially block its complement inhibitory activity, thereby increasing complement-mediated killing of larvae (Hao et al., 2014). Mice immunized with recombinant protein (Yang et al., 2010), epitope peptides (Wei et al., 2011; Gu et al., 2013, 2017), or the DNA (Wang et al., 2016, 2017) of Ts-Pmy were conferred with significant protection against the challenge of *T. spiralis* muscle larvae. Thus, Ts-Pmy has become the leading vaccine candidate for the control of trichinellosis. The RNAi-mediated silencing of Pmy expression in *T. spiralis* was also verified to reduce viability and infectivity of treated infective larvae (Chen et al., 2012).

## Conclusion

The complement system plays a major role in combating the establishment of invading pathogens, and acts as the first line of host defense against the invading parasites. Parasites have developed sophisticated mechanisms as survival strategies to defend against complement attack including recruitment of host regulatory proteins and expression of proteins either homologs to host regulators or directly blocking activated complement molecules to evade or inhibit complement activation. In this review, we analyzed a considerable number of proteins that are expressed or recruited by parasites and involved in the regulation of the host complement system as a strategy to escape host complement-mediated killing. Effective evasion of host complement attack is a crucial step for the survival of parasites within the host. Therefore, parasite-expressed CRPs have now become important targets for the development of drugs and vaccines against parasitic infections.

## Author Contributions

SS, XS, and XZ conceived the concept for this review article. SS and XS wrote the manuscript. SS constructed the table and figures. BZ, XZ, and YC read, edited, and reviewed the manuscript.

## Conflict of Interest Statement

The authors declare that the research was conducted in the absence of any commercial or financial relationships that could be construed as a potential conflict of interest.

## Abbreviations

APC: antigen presenting cells
C1-INH: C1 inhibitor
C4BP: C4b-binding protein
C8BP: C8-binding protein
CPN1: carboxypeptidase N
CRIg: complement receptor of the immunoglobulin family
CRIT: complement receptor inhibitor trispanning
CRP: complement regulatory protein
CRT: calreticulin
DAF: decay-accelerating factor
FB: factor B
FD: factor D
FH: factor H
FI: factor I
FP: properdin
GAPDH: glyceraldehyde-3-phosphate dehydrogenase
InsP6: myo-inositol hexakisphosphate
MAC: membrane attack complex
MASP: MBL-associated serine protease
MBL: mannose-binding lectin
MCP: membrane-cofactor protein
MSP: major surface protease
MSP1: merozoite surface protein 1
PAMPs: pathogen-associated molecular patterns
PfMSP3.1: *Plasmodium falciparum* merozoite surface protein 3 family
PfPIG-M: *P. falciparum* mannosyltransferase
Pmy: paramyosin
PRM: pattern recognition molecule
RCA: regulators of complement activation
SCIP-1: schistosome complement inhibitor-1
SMIPP-S: scabies mite proteolytically inactive serine protease paralog
SP40/40: S-protein
TOR: trispanning orphan receptor
